# Merkel cell polyomavirus Tumor antigens expressed in Merkel cell carcinoma function independently of the ubiquitin ligases Fbw7 and β-TrCP

**DOI:** 10.1371/journal.ppat.1007543

**Published:** 2019-01-28

**Authors:** Kristine N. Dye, Markus Welcker, Bruce E. Clurman, Ann Roman, Denise A. Galloway

**Affiliations:** 1 Division of Human Biology, Fred Hutchinson Cancer Research Center, Seattle, WA, United States of America; 2 Department of Global Health, University of Washington; Seattle, WA, United States of America; 3 Division of Clinical Research, Fred Hutchinson Cancer Research Center, Seattle, WA, United States of America; University of Michigan, UNITED STATES

## Abstract

Merkel cell polyomavirus (MCPyV) accounts for 80% of all Merkel cell carcinoma (MCC) cases through expression of two viral oncoproteins: the truncated large T antigen (LT-t) and small T antigen (ST). MCPyV ST is thought to be the main driver of cellular transformation and has also been shown to increase LT protein levels through the activity of its Large-T Stabilization Domain (LSD). The ST LSD was reported to bind and sequester several ubiquitin ligases, including Fbw7 and **β**-TrCP, and thereby stabilize LT-t and several other Fbw7 targets including c-Myc and cyclin E. Therefore, the ST LSD is thought to contribute to transformation by promoting the accumulation of these oncoproteins. Targets of Fbw7 and **β**-TrCP contain well-defined, conserved, phospho-degrons. However, as neither MCPyV LT, LT-t nor ST contain the canonical Fbw7 phospho-degron, we sought to further investigate the proposed model of ST stabilization of LT-t and transformation. In this study, we provide several lines of evidence that fail to support a specific interaction between MCPyV T antigens and Fbw7 or **β**-TrCP by co-immunoprecipitation or functional consequence. Although MCPyV ST does indeed increase LT protein levels through its Large-T Stabilization domain (LSD), this is accomplished independently of Fbw7. Therefore, our study indicates a need for further investigation into the role and mechanism(s) of MCPyV T antigens in viral replication, latency, transformation, and tumorigenesis.

## Author summary

Merkel cell carcinoma (MCC) is a very aggressive and deadly neuroendocrine skin cancer. Merkel cell polyomavirus (MCPyV) contributes to the development and maintenance of 80% of MCCs through the expression of its truncated large tumor antigen (LT-t) and small tumor antigen (ST). MCPyV ST is thought to be primarily responsible for transformation and tumorigenesis through many mechanisms including stabilization of MCPyV LT-t and other cellular proteins involved in proliferation such as c-Myc. As c-Myc is a known target substrate, and MCPyV LT-t is a proposed target substrate, of the ubiquitin ligase Fbw7, it is currently thought that ST stabilizes these proteins and transforms cells through binding and perturbing the function of Fbw7. However, neither MCPyV LT-t nor ST contain a canonical Fbw7 degron sequence necessary for this interaction. MCPyV LT-t, found in MCCs, does not bind to, nor is targeted by, Fbw7. However, an ill-defined, unidirectional interaction between MCPyV LT, ST, and Fbw7 was observed, but had no functional consequence. Therefore, this study calls for further investigation into the mechanism(s) by which MCPyV ST leads to the development and maintenance of MCC.

## Introduction

Merkel cell carcinoma (MCC) is an extremely rare, but aggressive, neuroendocrine skin cancer with an incidence of 0.7 cases/100,000 person-years in the United States, and a less than 45% 5-year survival rate, making MCC almost three times as lethal as melanoma [1, 2]. Although MCC was first described in 1972, it wasn’t until 2008 that a previously undescribed polyomavirus was found to be clonally integrated into 80% of MCC tumors and was thus termed Merkel cell polyomavirus (MCPyV) [3, 4]. MCPyV is a small, circular, dsDNA virus that utilizes alternative splicing of its early region (ER) to generate four ER proteins including the Large Tumor antigen (LT), Small Tumor antigen (ST), 57kT, and Alternate Large-T Open reading frame (ALTO) [5]. However, in MCCs only ST and a truncated form of the LT antigen (LT-t) are expressed. The tumor specific truncation of LT occurs as a consequence of a premature stop codon or deletion in the viral genome downstream of the Rb binding site and before the LT helicase domain [6]. Selection of this truncation is thought to be driven through the prevention of several activities and consequences deleterious for tumorigenesis [7, 8].

Unlike other oncogenic polyomaviruses, such as simian vacuolating virus 40 (SV40) and murine polyomavirus (MPyV), the dominant transforming protein of MCPyV appears to be ST; however, LT-t has also been found to be necessary for tumor maintenance through the activity of its Rb binding domain [9–11]. Several mechanisms of MCPyV ST mediated transformation have been proposed including, but not limited to, increased cap-dependent translation through 4E-BP1 phosphorylation [9, 12], a more migratory phenotype through dysregulation of actin and microtubule dynamics [13, 14], and transcriptome perturbations through binding of L-Myc and EP400 [15, 16].

It has also been proposed that MCPyV ST can perturb the functions of several cellular ubiquitin ligases, most notably SCF^Fbw7^, to accomplish transformation [12, 17–19]. SCF^Fbw7^ is a multi-protein complex responsible for binding and ubiquitinating target proteins for proteasomal degradation [20]. Fbw7 is the component of the SCF (Skp1, Cul1 and Fbox protein) ubiquitin ligases responsible for both substrate recognition and recruitment of the ubiquitination machinery. There are three isoforms of Fbw7 which localize to different subcellular compartments: Fbw7α is nucleoplasmic, Fbw7β is cytoplasmic, and Fbw7γ is nucleolar, with Fbw7α being thought to perform the majority of Fbw7 functions [21, 22]. All three Fbw7 isoforms contain three functional domains: 1) the Fbox domain binds Skp1, thereby linking Fbw7 to the SCF ubiquitination machinery, 2) the WD40 domain forms a **β**-propeller with two phosphate binding pockets that recognize target proteins containing phosphorylations within a conserved Cdc4 phospho-degron (CPD), named after the budding yeast Fbw7 homolog CDC4, and 3) the dimerization domain mediates the formation of Fbw7 dimers [20, 22–25] (S1A Fig). Most Fbw7 substrates share a dually phosphorylated degron with both phosphates spaced four amino acids apart (e.g. pTPPxpT/S), while the “+4” position can also be a negatively charged amino acid, and the “0” position is preceded by several hydrophobic amino acids (S1B Fig) [22]. Of note, this CPD sequence is conserved among all currently known Fbw7 targets [24]. Three arginine residues within the WD40 domain of Fbw7 (R465, R479, and R505) make direct contacts with degron phosphates and are most critical for substrate interactions [20, 25]. Fbw7 is frequently mutated in cancers due to its tumor suppressive function of regulating protein levels of several cellular oncoproteins such as c-Myc and cyclin E [21, 22, 26–28]. Interestingly, Fbw7 function has also been shown to be perturbed by the LT protein of SV40. SV40 LT contains a decoy-CPD at its extreme C-terminus (pTPPPE), which can directly interact with the WD40 domain of Fbw7 and consequently decrease turnover of normal Fbw7 target substrates (S1B Fig) [29].

In contrast to SV40 LT, Kwun et al. have proposed MCPyV LT to be targeted for proteasomal degradation through interaction with the WD40 domains of several ubiquitin ligases including Fbw7 and Fbw1 (**β**-TrCP) [17, 18]. As MCPyV LT and LT-t play an important role in viral replication and tumor maintenance, respectively, their rapid turnover would be deleterious [17, 18]. However, amino acids 91–95 of MCPyV ST have been shown to increase LT protein levels, and was thus termed the Large-T Stabilization Domain (LSD) [17]. Currently, the ST LSD is thought to increase LT protein levels by binding and sequestering several ubiquitin ligases, including Fbw7, from LT and their other cellular targets, such as c-Myc, thereby decreasing their turnover (S1C Fig) [17]. Such an activity, similar to SV40 LT, would suggest a possible role of the ST LSD in transformation and tumorigenesis, as increased concentrations of a MCPyV tumor antigen and cellular oncoproteins could lead to aberrant cellular proliferation, increased translation, and genomic instability [12, 17, 19]. Indeed, mutation of the LSD has been shown to ablate the ability of MCPyV ST expressing cells to form colonies in soft agar and promote epithelial hyperplasia in pre-term transgenic mouse embryos [17, 30]. Furthermore, as MCPyV LT is necessary for viral replication, the activity of the ST LSD has also been proposed to initiate viral replication and exit from viral latency [18].

In this study we further investigated the proposed interaction between MCPyV T antigens and Fbw7, as we find targeting of MCPyV LT by Fbw7 conceptually difficult to rationalize, and because of the absence of a canonical Fbw7 CPD in both MCPyV LT and ST. Contrary to the interactions and model proposed by Kwun et al., MCPyV LT, LT-t and ST failed to specifically interact with, or be destabilized by, Fbw7 and Fbw1 [17, 18]. Furthermore, the MCPyV ST LSD was capable of increasing LT protein levels independent of Fbw7. Therefore, this study calls for further investigation into the molecular mechanisms by which MCPyV ST contributes to the development and maintenance of MCPyV positive MCC.

## Results

### SV40 LT, but not MCPyV T antigens, co-immunoprecipitate with Fbw7α

**Fig 1:**
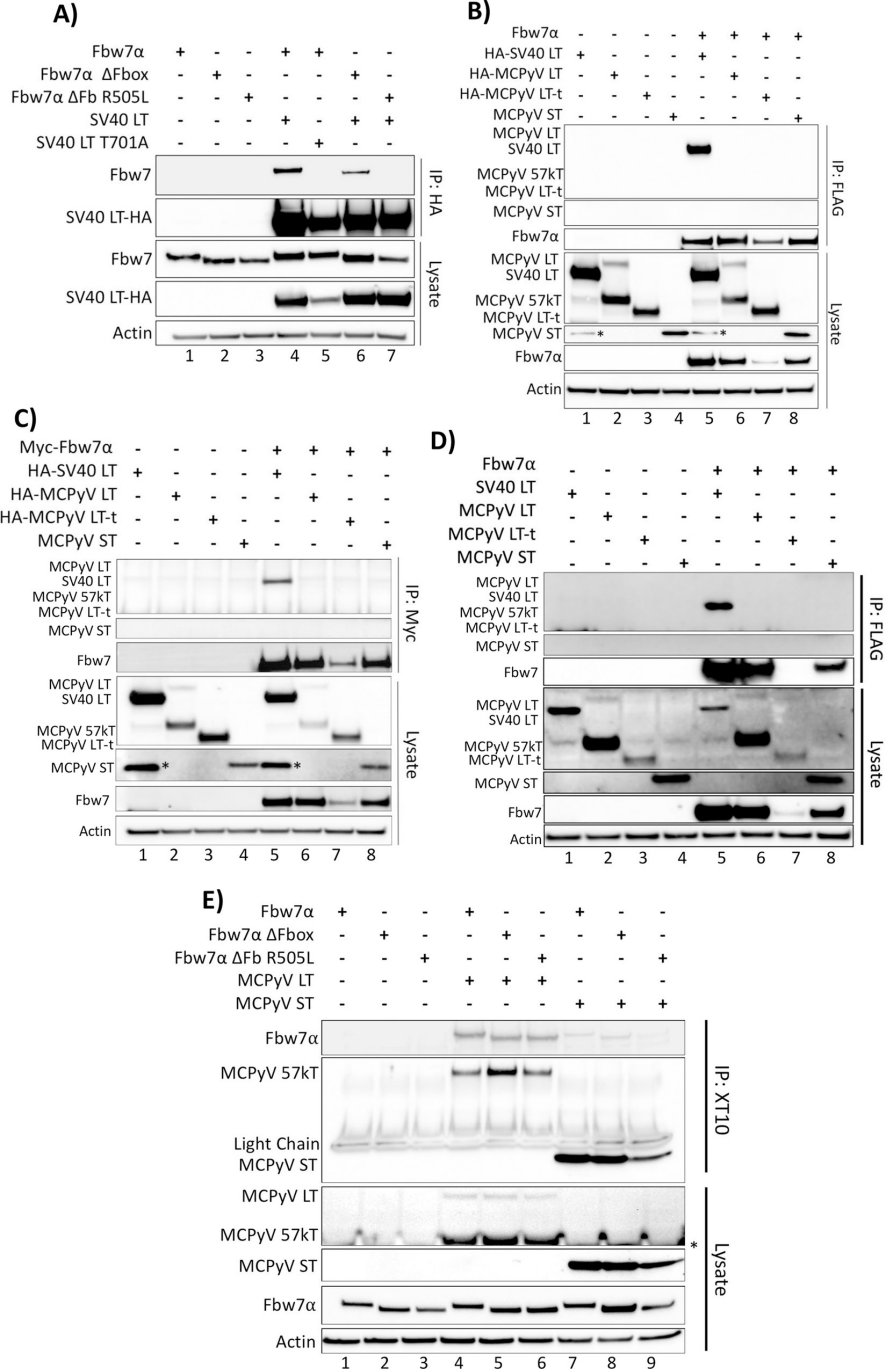
SV40 LT, but not the MCPyV T antigens, co-immunoprecipitate with Fbw7α. **(A)** The SV40 LT antigen was pulled down with an anti-HA antibody from whole cell lysates of 293A cells expressing individual or combinations of HA-SV40 LT or the T701A mutant (5μg), wild-type FLAG-Fbw7 (4.5μg), or FLAG-Fbw7 ΔFbox/R505L mutants (3μg). Detection of co-immunoprecipitated Fbw7 was performed by immunoprecipitating with anti-HA, followed by immunoblotting with anti-FLAG. **(B)** The reciprocal IP to Fig 1A was performed with HA-SV40 LT, the MCPyV T antigens (HA-LT (5μg), HA-LT-t (10.5μg), and untagged ST (1μg)) and Fbw7, in which Fbw7 was pulled down (FLAG) and immunoblotted for interacting T antigens (anti-HA/2T2 (common-T antibody)). **(C)** An identical co-immunoprecipitation as Fig 1B was performed, except Fbw7 with an N-terminal Myc tag was pulled down from cellular lysates using a Myc tag specific antibody (9E10). **(D)** An identical co-immunoprecipitation as Fig 1B was performed with untagged SV40 and MCPyV T antigens. SV40 and MCPyV T antigens were detected by XT10 immunoblotting. **(E)** A co-immunoprecipitation between MCPyV T antigens (LT and ST) and Fbw7 was also performed through pull-down of the T antigens (XT10—common-T antibody) and detection of co-immunoprecipitated Fbw7 (anti-FLAG). Asterisks (*) denote non-specific bands.

To first validate an interaction between Fbw7 and a well-established target, SV40 LT, co-immunoprecipitations were performed (Fig 1A and S1 Table) [29, 31, 32]. In 293A cells FLAG tagged Fbw7α was co-transfected with HA tagged SV40 LT and immunoprecipitated with an anti-HA antibody. As expected, Fbw7α readily co-immunoprecipitated with SV40 LT (Fig 1A, lane 4). SV40 LT was also found to co-immunoprecipitate the Fbw7α ΔFbox mutant, which is unable to ubiquitinate its substrate protein but is still capable of interacting with its substrate through an intact WD40 domain (Fig 1A, lane 6). The interaction between SV40 and Fbw7α also displayed degron dependence as the SV40 LT CPD mutant, in which the central phosphorylated threonine of the CPD was substituted with an alanine, was incapable of co-immunoprecipitating wild-type Fbw7 (Fig 1A, lane 5). Additionally, the interaction was disrupted by an Fbw7 WD40 mutant, in which a crucial arginine of the Fbw7 WD40 domain is mutated to a leucine (Fbw7α ΔFb R505L) (Fig 1A, lane 7).

Having confirmed the reliability of the immunoprecipitation (IP) protocol utilized, the interaction between the CPD deficient MCPyV T antigens and Fbw7 was investigated. 293A cells were co-transfected with either HA-tagged MCPyV full-length LT, MCPyV truncated LT (LT-t), untagged MCPyV ST, or HA-SV40 LT, in combination with Fbw7α. Despite the fact that 293A cells express adenovirus E1A and E1B proteins, they were chosen as our experimental model to replicate the studies done by Kwun et al. [17, 18] and other Fbw7 substrate studies [21, 29]. Fbw7 proteins were pulled-down with an antibody recognizing a FLAG tag on the N-terminus of Fbw7, reciprocal to the IP performed in Fig 1A. Surprisingly, only SV40 LT co-immunoprecipitated with Fbw7α in this IP, consistent with it being a bona-fide Fbw7 binding partner, whereas none of the MCPyV T antigens (LT, LT-t, and ST) interacted with Fbw7α even though their expression was confirmed in the cellular lysates (Fig 1B). It should be noted that the alternatively spliced form of the full-length MCPyV LT, 57kT, was preferentially pulled-down in these assays; however, neither full-length LT nor 57kT are expressed in MCC tumors [6]. Also of note, the tumor expressed MCPyV LT-t was found to decrease Fbw7α protein levels, as seen in the protein lysate (Fig 1B, lane 7). This decrease was not found to be a result of LT-t decreasing Fbw7α transcription by qRT-PCR (S2A Fig). Currently, the mechanism underlying this observation is not understood, and could either be a physiologically irrelevant consequence of artificial LT-t expression, or lead to the discovery of a novel role for MCPyV LT-t.

To ensure that the inability of FLAG tagged Fbw7 to pull-down the MCPyV T antigens is not a consequence of the tag used, we performed an identical experiment using Myc tagged Fbw7. Consistent with Fig 1B, Myc tagged Fbw7 was capable of co-immunoprecipitating SV40 LT, but not the MCPyV T antigens (Fig 1C), suggesting that the tag used to pull-down Fbw7 is not responsible for the inability to observe an interaction between the MCPyV T antigens and Fbw7.

It is possible that the HA tags found on MCPyV LT and LT-t could pose a steric hindrance to the interaction with Fbw7, although this would not explain the inability of MCPyV ST to bind Fbw7, as ST was untagged in these experiments. A similar co-immunoprecipitation to Fig 1B was performed by transfecting 293A cells with FLAG tagged Fbw7, and untagged SV40 and MCPyV T antigens, followed by pulldown of Fbw7 and XT10 immunoblotting to detect co-immunoprecipitated T antigens. Again, untagged SV40 LT was capable of co-immunoprecipitating with Fbw7; however, none of the untagged MCPyV T antigens co-immunoprecipitated (Fig 1D). Therefore, the N-terminal HA tag is not responsible for the inability of the MCPyV T antigens to co-immunoprecipitate with Fbw7.

In an effort to understand the discrepancies between our data and those of Kwun et al. [17, 18], a reciprocal co-immunoprecipitation was performed with an antibody that recognizes the J-domain of both SV40 and MCPyV T antigens, XT10. Immunoblotting for Fbw7 (FLAG) revealed an interaction between MCPyV LT and Fbw7α, and a very weak interaction between MCPyV ST and Fbw7α (Fig 1E). Furthermore, we were surprised to find that MCPyV LT and ST bound equivalently to wild-type Fbw7α and the Fbw7α WD40 mutant, R505L (Fig 1E, lanes 6 and 9), which is unable to bind any other known Fbw7 substrates, including SV40 LT (Fig 1A, lane 7). Although the interaction between MCPyV ST and Fbw7 R505L appears to be slightly fainter than the already weak interaction between Fbw7 and ST, this is most likely a consequence of less ST pulled-down in this lane (Fig 1E, lane 9).

To determine whether XT10 was responsible for the contradictory binding results between co-immunoprecipitations performed in different directions, immunoprecipitations were performed with additional antibodies specific for the region shared by MCPyV LT, LT-t, 57kT and ST (common T–Ab5) (S3A Fig), or LT, 57kT and LT-t only (CM2B4, Ab3) (S3B Fig) (S1D Fig). MCPyV LT co-immunoprecipitated Fbw7 with each MCPyV T antigen antibody used for the pulldown (S3A and S3B Fig, lane 3). Similarly, MCPyV ST again very weakly co-immunoprecipitated Fbw7 (S3A Fig, lane 7). As seen with XT10 pull-down (Fig 1E), LT was found to co-immunoprecipitate with the Fbw7 WD40 mutant (Fbw7 ΔFb R505L) (S3A and S3B, lane 5). Taken together, it appears that MCPyV LT and ST interact with Fbw7 nonspecifically when pulled-down with antibodies specific for the MCPyV T antigens, and this interaction is not reproducible when pulled-down with an Fbw7 anti-tag antibody (FLAG or Myc).

### MCPyV ST increases LT protein levels independently of Fbw7, but dependent on the LSD

**Fig 2:**
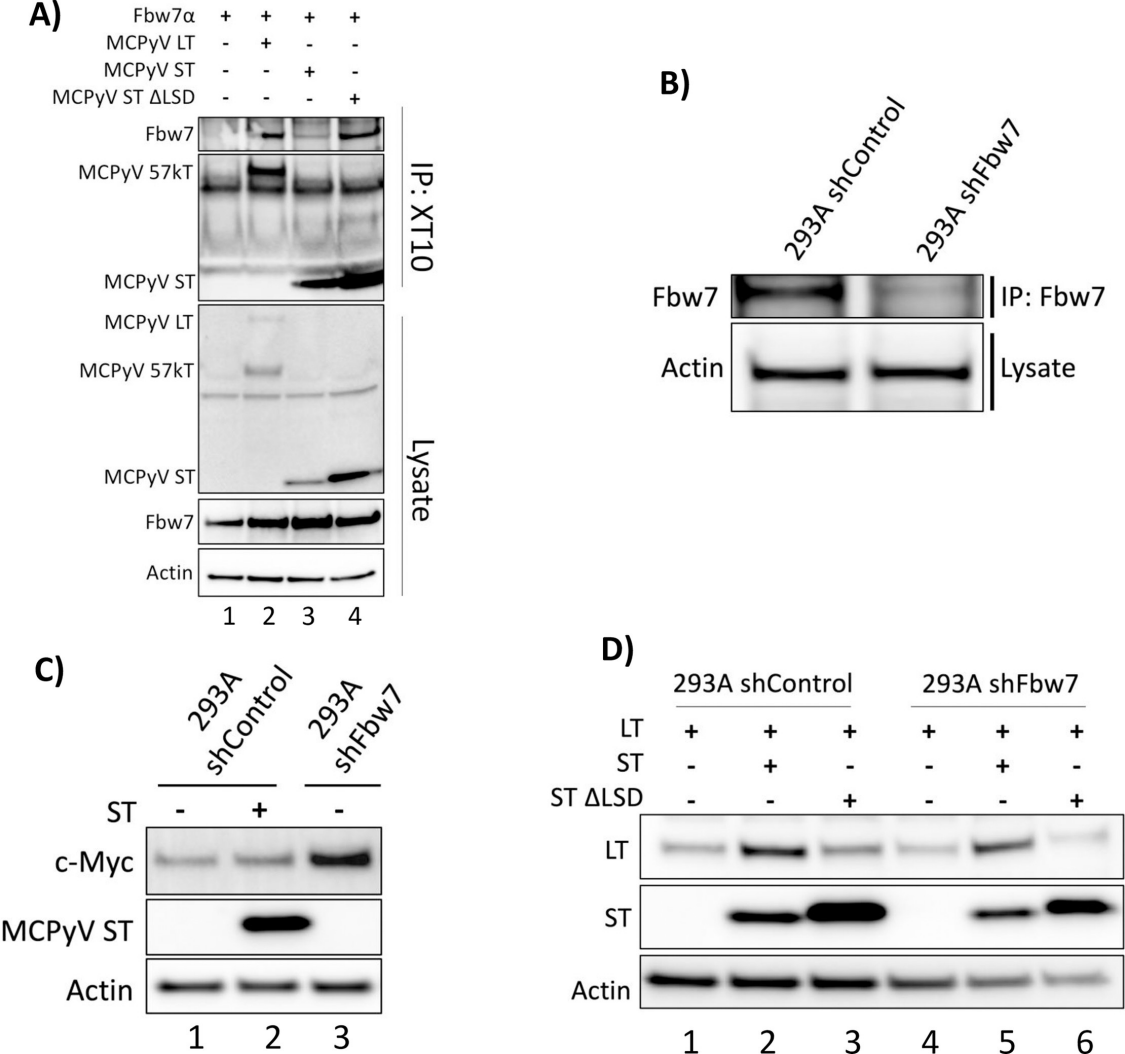
MCPyV ST increases LT protein levels independently of Fbw7, but dependent on the LSD. **(A)** A co-immunoprecipitation between MCPyV T antigens (LT, ST, ST ΔLSD) and Fbw7 was performed through pull-down of the T antigens (XT10—common-T antibody) and detection of co-immunoprecipitated Fbw7 (anti-FLAG). **(B)** Fbw7 knockdown was confirmed in 293A cells transduced with lentiviruses containing shControl or shFbw7 by pulling down and immunoblotting for Fbw7 (A301-720). **(C)** Endogenous c-Myc protein levels were assessed by western blot in 293A expressing shControl, shFbw7, and/or MCPyV ST. **(D)** MCPyV LT protein levels were assessed in control and Fbw7 knockdown 293A cells with and without ST or ST ΔLSD co-expression.

The LSD of MCPyV ST has been proposed to be responsible for binding Fbw7α even though this domain does not contain a recognizable conserved CPD (S1B Fig) [17]. To assess the role of the ST LSD in the weak interaction between ST and Fbw7, a ST ΔLSD mutant was constructed and tested in similar co-immunoprecipitation experiments. In our hands, the ST ΔLSD mutant was still capable of interacting with Fbw7, and appeared to bind more strongly to Fbw7α than wild-type ST, most likely a consequence of greater amounts of ST ΔLSD mutant being expressed (Fig 2A, lane 4). Similar results were found when the ST ΔLSD mutant was immunoprecipitated with Ab5 (S3A Fig, lane 8).

The current model proposes MCPyV ST preferentially binds and sequesters Fbw7 away from MCPyV LT and its other cellular targets, suggesting ST has a higher affinity for Fbw7 than LT (S1C Fig) [17, 18]. However, since the unidirectional interaction between MCPyV ST and Fbw7α was found to be weaker than that of LT (Figs 1E and 2A, S3A Fig), and independent of the ST LSD (Fig 2A, S3A Fig), we examined whether ST was capable of increasing MCPyV LT protein levels in the absence of Fbw7. Knockdown of Fbw7 in 293A cells was performed with lentiviruses containing either an shControl or shFbw7, and the knockdown was confirmed by pulldown and immunoblotting of endogenous Fbw7 (Fig 2B). To investigate the described perturbation of Fbw7 by MCPyV ST, a downstream target of Fbw7, c-Myc, was analyzed in 293A cells expressing shControl, shFbw7, and/or MCPyV ST. As expected, Fbw7 knockdown led to increased c-Myc protein levels (Fig 2C, lane 3); however, MCPyV ST expression in shControl 293A cells did not increase endogenous c-Myc protein levels (Fig 2C, lane 2). Therefore, MCPyV ST’s inability to stabilize a downstream Fbw7 target, c-Myc, is contrary to the model of ST sequestration of Fbw7. As has been described previously, MCPyV LT protein levels were increased by co-expression of MCPyV ST, but not the ST ΔLSD mutant (Fig 2D, lanes 1–3) [17]. However, in Fbw7 knockdown cells MCPyV ST also increased LT protein levels (Fig 2D, lane 5). Therefore, the LSD sequence in ST is capable of increasing LT protein levels independent of Fbw7, supporting the lack of functional significance of the LSD sequence in the unidirectional interaction between MCPyV ST and Fbw7. Taken together, MCPyV ST does not perturb the function of Fbw7, and thus opens the door for further investigation into the mechanism by which the ST LSD increases LT protein levels.

### MCPyV LT-t is not bound or destabilized by Fbw7

**Fig 3:**
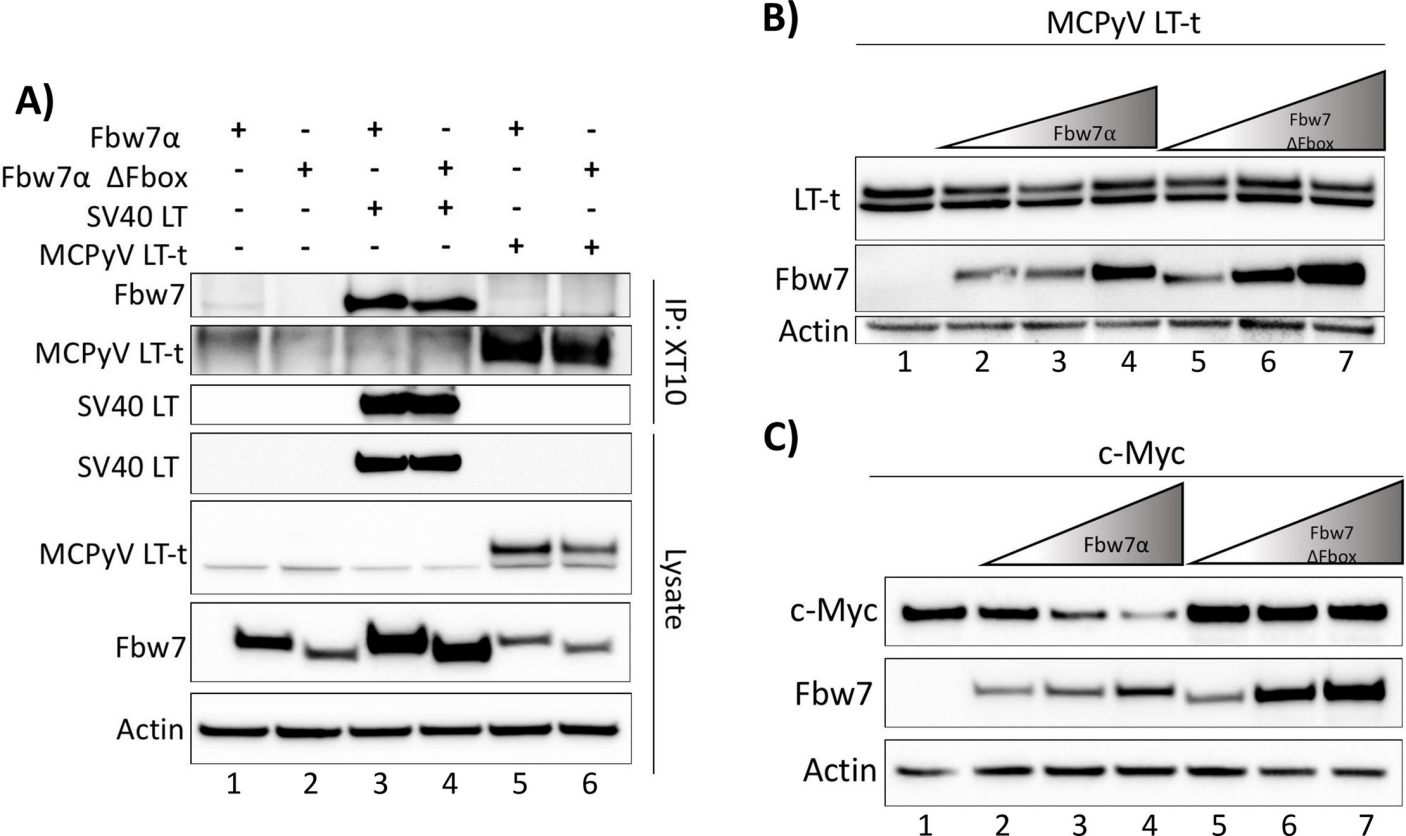
MCPyV LT-t is not bound or destabilized by Fbw7. **(A)** Whole cell lysates of 293A cells transfected with individual or combinations of FLAG-Fbw7 (4.5μg), ΔFbox (3μg), HA-SV40 LT (5μg), MCPyV LT-t (10.5μg), were pulled-down with XT10, and immunoblotted with anti-FLAG. **(B)** 293A cells were transfected with a constant amount of MCPyV LT-t (10μg) and increasing amounts of Fbw7 (2.5μg, 5μg, 10μg) or the stable, degradation incompetent, Fbw7 ΔFbox (1.25μg, 2.5μg, 5μg). MCPyV LT-t protein levels were compared by 2T2 immunoblotting (common-T antibody). **(C)** Similar to Fig 3B, 293A cells were transfected with consistent amounts of HA-c-Myc (2μg) and increasing amounts of Fbw7 (0.1μg, 0.25μg, and 0.5μg). c-Myc protein levels were compared through detection of c-Myc by immunoblotting with anti-HA.

In MCPyV positive MCC only ST and LT-t are expressed [4, 6]. Since ST sequestration of Fbw7α away from LT-t is a proposed mechanism of ST induced transformation and tumorigenesis [17], we next sought to determine whether LT-t is bound and destabilized by Fbw7α. MCPyV LT-t or SV40 LT were immunoprecipitated with either the XT10, Ab3, or Ab5 antibodies from whole cell lysates of 293A cells transfected with the T antigens and Fbw7α. Unlike SV40 LT, MCPyV LT-t was unable to co-immunoprecipitate with Fbw7α (Fig 3A, lane 5; S3A and S3B Fig, lane 6). The interaction between MCPyV LT-t and Fbw7α has been described as extremely transient due to rapid degradation and is, therefore, only observable after treatment with the broad proteasome inhibitor MG132 [17]. Similar to Kwun et al., we were able to see an interaction between MCPyV LT-t and Fbw7α when treated with MG132; however, an even stronger interaction was observed between our negative control, SV40 LT-T701A, and Fbw7α with MG132 treatment, suggesting that pleiotropic effects of MG132 treatment can lead to false positives (compare Fig 1A with S2B Fig) [17]. A more direct and reliable way to assess binding partners of Fbw7α uncoupled from turnover is through the utilization of the degradation incompetent Fbw7α ΔFbox mutant. MCPyV LT-t was also unable to co-immunoprecipitate with Fbw7α ΔFbox, further suggesting the inability of these two proteins to interact, even in the absence of turnover (Fig 3A, lane 6).

To determine if MCPyV LT-t is destabilized by co-expression of Fbw7α, cells were co-transfected with identical amounts MCPyV LT-t and increasing amounts of either Fbw7α or the degradation incompetent Fbw7α ΔFbox mutant. Consistent with Fbw7α not binding LT-t, co-expression of Fbw7α did not reduce LT-t protein levels (Fig 3B). This is in contrast to c-Myc, which was readily destabilized by Fbw7α, but was not destabilized by the Fbw7α ΔFbox mutant (Fig 3C). Together, these results suggest that the LT-antigen found in MCC, LT-t, is neither bound nor destabilized by Fbw7α.

### MCPyV LT unidirectionally binds Fbw7 through an unidentified domain

**Fig 4:**
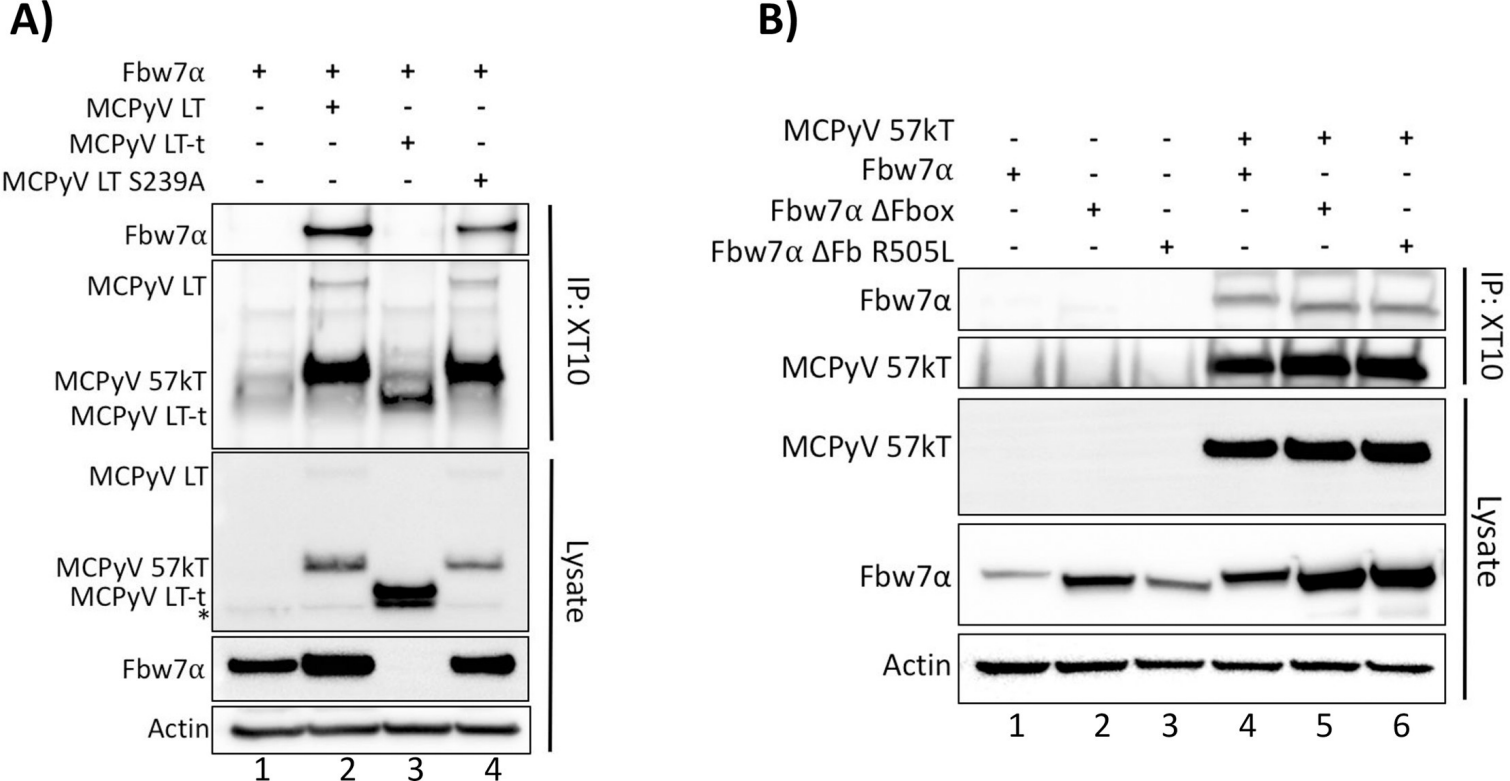
MCPyV LT and 57kT bind Fbw7α independently of its WD40 domain and MCPyV LT S239. **(A, B)** MCPyV LT was pulled-down with XT10 from whole cell lysates of 293A cells transfected with individual or combinations of full-length MCPyV LT S239A (5μg), MCPyV LT-t (10.5μg), MCPyV 57kT (5μg), Fbw7 (4.5μg), ΔFbox (3μg), or R505L (3μg), and immunoblotted with anti-FLAG to detect co-immunoprecipitated Fbw7. **(B)** A plasmid encoding only the sequence of MCPyV 57kT was similarly pulled-down and co-immunoprecipitated Fbw7 was detected. Asterisks (*) denote non-specific bands.

Kwun et al. mapped the domain of MCPyV LT-t targeted by Fbw7α to residues around S239, although this domain does not resemble a canonical Fbw7 CPD (S1B Fig) [18]. Because we did not detect an interaction between LT-t and Fbw7, the S239A mutation was constructed in full-length LT; however, this mutant was still capable of co-immunoprecipitating Fbw7α (Fig 4A; S3A and S3B Fig, lane 4). Therefore, since MCPyV LT does not contain a canonical degron, and the proposed degron, S239, was found to be dispensable, it is unknown how or where the unidirectional interaction between MCPyV LT and Fbw7 is occurring.

The MCPyV T antigen specific antibodies used in these immunoprecipitations can pull-down both MCPyV LT and 57kT since they are identical in sequence with the exception of a spliced-out intron of the 57kT (S4A Fig). Therefore, it is unclear whether or not the observed interaction between MCPyV LT/57kT and Fbw7α is specific to MCPyV LT, 57kT, or a domain shared by both. However, a construct that could only express the 57kT also readily co-immunoprecipitated with Fbw7 independent of the WD40 domain (Fig 4B).

Since MCPyV LT and 57kT, but not LT-t, were capable of binding Fbw7α, the Fbw7α binding domain of MCPyV LT and 57kT must reside in the C-terminal 100 amino acids common to both proteins (S4A Fig). Although the decoy-CPD of SV40 LT is found at its extreme C-terminus [29], the entire full-length MCPyV LT does not contain a canonical Fbw7 CPD. To identify the domain within the C-terminal 100 amino acids of MCPyV LT responsible for binding Fbw7α, an alanine scan in which sequential 5 amino acids were substituted with alanines was performed. Every stable mutant of the alanine scan was capable of co-immunoprecipitating Fbw7α (S4B–S4E Fig), though we cannot rule out interactions with sequences that when mutated destabilized LT. It is also possible that there are multiple weak degrons in the C-terminus of MCPyV LT, though this type of binding has been refuted [33, 34] and would nevertheless be inconsequential to MCPyV transformation as this region of LT is not expressed in MCCs. Therefore, the MCPyV LT domain responsible for the unidirectional interaction observed between LT and Fbw7α is still unresolved.

### The site on Fbw7 to which binding of MCPyV T antigens occurs is unknown

**Fig 5:**
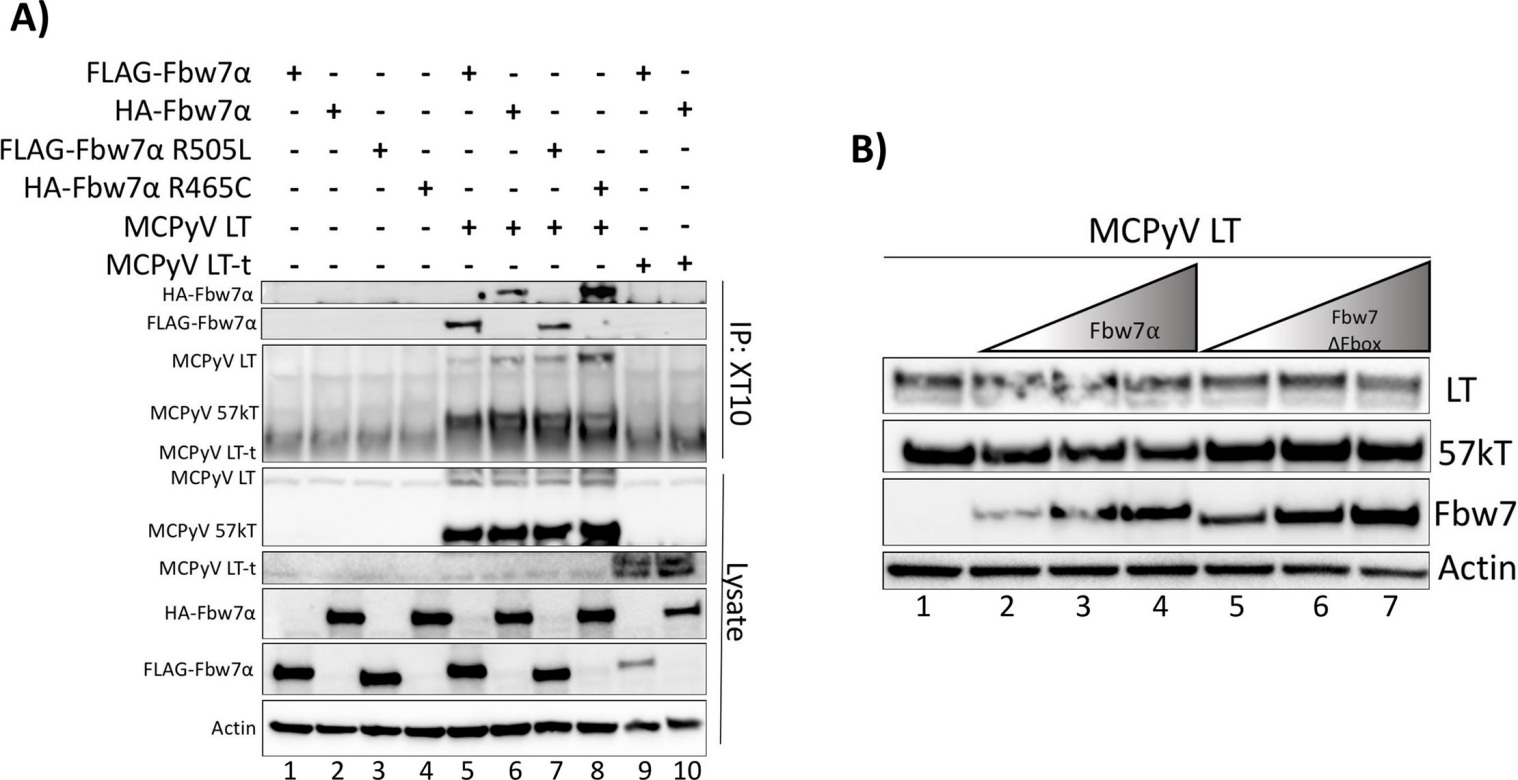
MCPyV LT is not bound or destabilized by the WD40 domain of Fbw7. **(A)** MCPyV LT was pulled-down from whole cell lysates of 293A cells transfected with MCPyV LT (5μg), LT-t (10.5μg) and either the FLAG-Fbw7α (4.5μg), or HA-Fbw7α construct (8μg). In addition, the FLAG-tagged WD40 mutant (FLAG-Fbw7α-R505L) (3μg), and the HA-tagged WD40 mutant provided by Kwun et al. (HA-Fbw7α-R465C) (8μg) were tested for their ability to co-immunoprecipitate with MCPyV LT and LT-t (17). **(B)** Consistent amounts of MCPyV LT (5μg) were co-expressed with increasing amounts of Fbw7 (2.5μg, 5μg, 10μg) or the degradation incompetent Fbw7 ΔFbox mutant. Immunoblotting with 2T2 was performed to compare MCPyV LT protein levels in each condition.

After unsuccessful identification of the MCPyV LT domain responsible for binding Fbw7α, we decided to also investigate the domain of Fbw7α responsible for binding MCPyV LT. As was shown in Fig 1E, MCPyV LT and ST were capable of interacting with an Fbw7α WD40 domain mutant, R505L, which has not been reported for any other known Fbw7 substrates. In contrast, the proposed interaction between MCPyV LT and Fbw7α was reported to be WD40 dependent, as an R465C mutation ablated this interaction [17]. Since all three arginine residues (R465, R479, R505) are necessary for the function of the Fbw7α WD40 domain, experiments were conducted to determine whether an Fbw7 R465C mutation would interfere with binding to LT. HA-tagged Fbw7α and the mutant R465C (gifts from Patrick Moore and Yuan Chang) in addition to FLAG-tagged Fbw7α and R505L were studied for their ability to interact with MCPyV LT or LT-t. Both wild-type Fbw7α constructs, regardless of having an HA or FLAG tag, were able to co-immunoprecipitate with MCPyV LT, but not the tumor-specific LT-t with XT10 pull-down (Fig 5A, lanes 5,6, 9, and 10). However, we found both WD40 domain mutants, R505L and R465C, to co-immunoprecipitate with MCPyV LT, further supporting that the unidirectional interaction observed between MCPyV LT and Fbw7 occurs independently of residue R465 in the WD40 domain (Fig 5A, lanes 7 and 8) [17]. Furthermore, the interaction between Fbw7α and MCPyV LT did not lead to destabilization of LT (Fig 5B). Thus, the interaction observed between MCPyV LT and Fbw7α is independent of the WD40 domain, and is consistent with the inability of Fbw7 to degrade LT, as the WD40 domain is responsible for positioning the substrate for ubiquitination and subsequent degradation [20, 22, 25].

The region of Fbw7α responsible for binding MCPyV LT mapped to the common C-terminal region shared by all Fbw7 isoforms (S5A and S5B Fig), which is known to contain three functional domains: the dimerization, Fbox, and WD40 domains [22]. As shown thus far, the Fbw7 Fbox and WD40 domains are dispensable for this interaction (Figs 1E,4B and 5A, S3A and S3B Fig); therefore, the dimerization domain was also assessed. A double Fbw7α mutant containing a deletion of both the Fbox and dimerization domain (Fbw7α ΔFD) was still capable of co-immunoprecipitating with both MCPyV LT and ST (S5C Fig). Therefore, it is unclear which domain of Fbw7α is responsible for the unidirectional interaction observed with MCPyV LT and ST.

### MCPyV LT-t and ST do not interact with the E3 ubiquitin ligase Fbw1 (β-TrCP)

**Fig 6:**
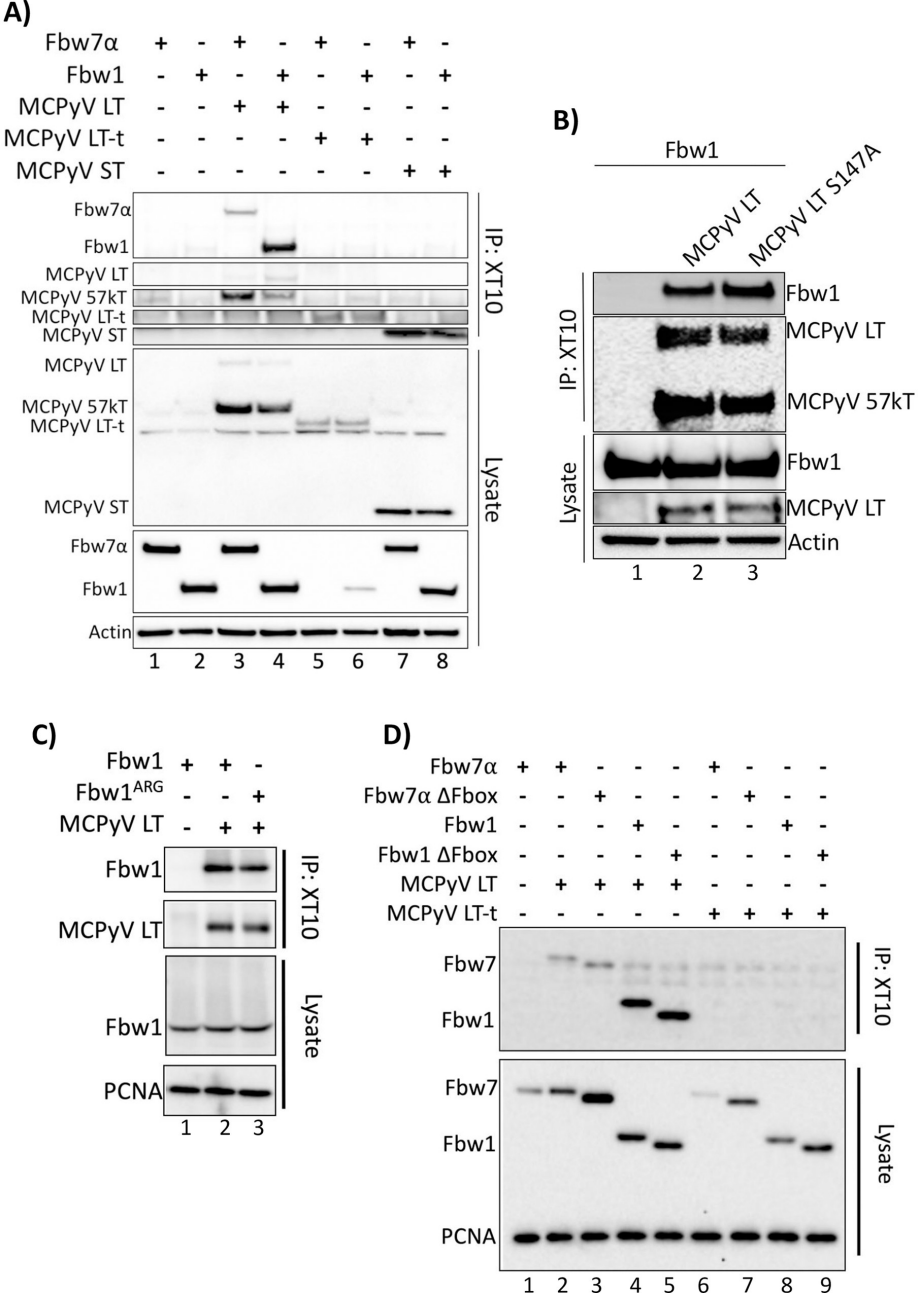
MCPyV LT-t and ST do not interact with the E3 ubiquitin ligase Fbw1 (β-TrCP). **(A)** Fbw1, was assessed for its ability to co-immunoprecipitate MCPyV T antigens. 293A cells co-transfected with individual or combinations of Fbw7 (4.5μg), Fbw1 (3μg), MCPyV LT (5μg), MCPyV LT-t (10.5μg), or MCPyV ST (1μg). MCPyV T antigens were pulled down with XT10, and Fbw7 or Fbw1 co-immunoprecipitation was detected by immunoblotting with anti-FLAG. **(B)** A mutation in the proposed Fbw1 binding domain of MCPyV LT-t, S147A, was also assessed for its ability to co-immunoprecipitate Fbw1. **(C and D)** MCPyV LT and LT-t were immunoprecipitated with XT10 and stained for co-immunopreciptated Fbw7, Fbw1, ΔFbox, or WD40 (Fbw1^ARG^) mutants (FLAG).

In addition to Fbw7, it has also been proposed that MCPyV LT is targeted for destruction by several other ubiquitin ligases including another Fbox containing E3 ubiquitin ligase, Fbw1(**β**-TrCP) [18]. The phospho-degron for Fbw1 recognition is also well defined and contains the sequence DpSGXXpS/pT [35], which is present at the extreme C-terminus of MCPyV LT. However, the reported domain of MCPyV LT responsible for the interaction with Fbw1 was mapped to S147 [18], which is missing several components of the canonical Fbw1 phospho-degron sequence (S1B Fig). Further, it has been hypothesized that MCPyV ST promiscuously binds several E3 ubiquitin ligases through its LSD to facilitate LT stability, including Fbw1 [12, 17–19], despite the fact that the ST LSD also shares no resemblance to the canonical Fbw1 degron (S1B Fig). To investigate the proposed interaction between MCPyV LT, LT-t, ST and Fbw1, co-immunoprecipitations were performed in which the T antigens were pulled-down and co-immunoprecipitated Fbw7 or Fbw1 was detected. MCPyV LT bound both Fbw7α and Fbw1; however, LT-t and ST failed to bind Fbw1 (Fig 6A). Furthermore, an alanine MCPyV LT mutant, S147A [18], was still capable of co-immunoprecipitating Fbw1 (Fig 6B). Similar results were found when the immunoprecipitation was performed with additional MCPyV T antigen specific antibodies (S6A and S6B Fig). Similar to Fbw7, MCPyV LT was capable of binding to both Fbw1 WD40 and ΔFbox mutants (Fig 6C lane 3, 6D lane 5), again suggesting the interaction between MCPyV T antigens and Fbw1 to be non-specific. Of note, MCPyV LT-t was also capable of decreasing Fbw1 protein levels, as was seen with Fbw7, (compare Fig 1B to Fig 6A) even though MCPyV LT-t was not found to bind either proteins. Therefore, this either novel function of MCPyV LT-t, or irrelevant consequence of artificial LT-t expression, is broadly acting.

## Discussion

Integration and expression of MCPyV T antigens accounts for 80% of MCC cases [4]; therefore, elucidating the mechanisms by which LT-t and ST accomplish transformation and tumorigenesis is paramount for the design of novel therapeutics to treat MCPyV positive MCCs. Furthermore, such insight could also have implications not only for understanding polyomavirus oncogenesis and disease, but also for cellular pathways, homeostasis, and broad disease pathology. MCPyV ST has been shown to increase protein levels of MCPyV LT, LT-t, and several other cellular oncoproteins involved in proliferation, such as c-Myc and cyclin E [17, 30]. This has been proposed to be a consequence of ST sequestration of several ubiquitin ligases, including Fbw7 and **β**-TrCP, and underlies the proposed mechanisms of ST enhancement of viral replication, and induction of transformation and tumorigenesis (S1C Fig) [12, 17–19].

Since many cellular pathways, processes, and fates are sensitive to, and directed by, the amount of a given protein, and ubiquitin-mediated proteolysis is irreversible, this process is tightly regulated [36]. To be targeted by Fbw7, and subsequently ubiquitinated for proteasomal degradation, a protein must 1) contain a specific and highly conserved amino acid sequence called a CPD, and 2) include multiple site-specific phosphorylations performed by other tightly regulated kinases within the cell [20, 25–27]. For these reasons, it was surprising that both MCPyV LT-t and ST have been reported to bind Fbw7, as neither viral protein contain the well-known, conserved, Fbw7 CPD.

The concept of viral oncoprotein perturbation of ubiquitin ligases is well-established [29, 37–41]. Although evolutionarily related, several significant differences lie between the interaction of SV40 LT and Fbw7, and the proposed interaction between MCPyV LT-t and Fbw7. SV40 LT binds the WD40 domain of Fbw7 through its canonical CPD sequence, leading to the stabilization of SV40 LT and Fbw7 targets, and mislocalization of the nucleolar Fbw7γ isoform [29]. In contrast, MCPyV LT-t has been proposed to also bind the WD40 domain of Fbw7, leading to its destruction despite the absence of a canonical CPD [17, 18]. While not impossible, it is difficult to rationalize how MCPyV LT-t is binding to the WD40 domain of Fbw7 in the absence of a CPD. Also, why MCPyV LT-t, a protein necessary for tumor viability, would retain sequences that would ultimately lead to its destruction, and therefore rely on the activities of another viral protein, ST, to avoid degradation is counter-intuitive [17, 18].

The interaction between MCPyV T antigens and ubiquitin ligases have been implicated in regulating both viral latency in normal, asymptomatic, viral infection (LT and ST), and transformation in the setting of MCC (LT-t and ST) [17, 18]. In this report, we provide several lines of evidence that oppose both proposed models.

It has been proposed that MCPyV ST promiscuously binds and sequesters several E3 ubiquitin ligases through its LSD; however, as each of these ubiquitin ligases recognize distinct phospho-degrons, it is difficult to imagine how one domain could interact with several different highly specific proteins [12, 17, 18, 20, 35]. In our hands, weak ST binding to Fbw7 was only observed unidirectionally and independent of the Fbw7 WD40 and ST LSD domains. Moreover, an interaction between MCPyV ST and Fbw1 was never observed. Therefore, we hypothesized that MCPyV ST, which lacks a canonical CPD and does not bind to the WD40 domain of Fbw7, may possess an alternative mechanism for binding and perturbing the function of Fbw7 independent of the WD40 domain. For instance, binding to another component of the SCF complex, as has been proposed for the adenovirus E1A protein [41], could explain the proposed ubiquitin ligase binding promiscuity of MCPyV ST. However, this hypothesis is also not supported by our data, as MCPyV ST also unidirectionally co-immunoprecipitated the Fbw7 ΔFbox mutant, which cannot recruit the remaining SCF complex. Furthermore, others have hypothesized that MCPyV ST may inhibit the formation of dimers to perturb Fbw7 function, as the dimerization domain of Fbw7 has been found to enhance binding to low affinity substrates [17, 23]. However, as we have also shown MCPyV ST capable of binding a dimerization domain Fbw7 mutant, it is unlikely that MCPyV ST utilizes this mechanism to perturb Fbw7 targeting of low affinity substrates, such as LT. Therefore, thorough investigation of several mechanisms by which MCPyV ST could perturb the function of ubiquitin ligases failed to uncover or support the proposed promiscuity of MCPyV ST ubiquitin ligase perturbation.

The proposed model of MCPyV ST mediated transformation suggests that MCPyV LT-t and other cellular oncoproteins are stabilized through MCPyV ST sequestration of Fbw7, leading to aberrant cellular proliferation and transformation [17]. Although our studies confirmed the ability of ST to increase LT protein levels through the LSD, this was accomplished independently of Fbw7. This is supported by the fact that MCPyV LT-t was never found to interact with, or be destabilized by, Fbw7. This suggests that MCPyV LT-t is not targeted by Fbw7, and is consistent with MCPyV ST not specifically binding to, or perturbing the function of Fbw7 to increase LT-t protein levels. Furthermore, MCPyV ST had no effect on c-Myc protein levels, a bona-fide Fbw7 substrate, further confirming no association between MCPyV ST and Fbw7. Thus, it can be concluded that the mechanism(s) by which ST increases LT-t protein levels, and transforms cells, does not involve sequestration and/or perturbation of Fbw7.

MCPyV LT destabilization by ubiquitin ligases has been proposed to play a role in maintaining viral latency in a normal infection, as reduced LT protein levels decreases viral replication [18]. Unlike tumor expressed MCPyV LT-t, we were able to observe a weak interaction between MCPyV LT and Fbw7; however, this interaction was only observed unidirectionally, was independent of proposed LT binding domains and the Fbw7 WD40, did not lead to destabilization of LT, and has no relevance to transformation and tumorigenesis. Although neither MCPyV LT nor ST contain a canonical Fbw7 CPD, one could hypothesize that the unidirectional interaction observed between LT, ST and Fbw7 could occur through a novel degron sequence found on these T antigens. However, such an interaction would still be dependent on an intact Fbw7 WD40 domain, which we found to be dispensable for binding. In addition, we were unable to identify the domains of the T antigens nor Fbw7 responsible for the unidirectional interaction through both direct mutagenesis of the proposed binding domains and comprehensive mutational screens. Thus, the proposed model of ST sequestration of ubiquitin ligases to stabilize LT protein levels and induce viral replication in the setting of a normal viral infection can not be confirmed at the level of the interaction itself or functionally.

To directly evaluate the discrepancies between our data and those of Kwun et al., plasmids were exchanged and tested in parallel. Here, both Fbw7 expressing constructs were capable of co-immunoprecipitating with MCPyV LT, but not the tumor expressed MCPyV LT-t. Also contrary to previous reports, the interactions observed between MCPyV LT and the provided Fbw7 constructs were independent of the WD40 domain. Thus, it is unlikely that the observed weak, unidirectional, destabilization and CPD/WD40 independent interaction observed between MCPyV T antigens and Fbw7 is relevant to the role of the T antigens in either the viral life-cycle or tumorigenesis.

In conclusion, we thoroughly investigated the specific interaction and downstream consequences of the proposed involvement of MCPyV T antigens and Fbw7. A likely artifactual interaction between MCPyV LT, ST and Fbw7 was observed, but not with LT-t, thereby dismissing the relevance of the proposed model in MCC tumorigenesis. It should be noted that ST is capable of increasing LT protein levels through its LSD; however, the mechanism by which this is accomplished by MCPyV ST is not fully understood, as Fbw7 was found to play no role in this interaction. In conclusion, we propose MCPyV ST to be capable of transformation and tumorigenesis independent of ubiquitin ligase perturbation, and therefore open the door for further investigation into the mechanisms by which MCPyV ST and the ST LSD contribute to viral replication, latency, and induce transformation and tumorigenesis.

## Materials and methods

### Cell culture, transfections, and lentiviral shRNA knock-downs

Adenovirus-transformed human embryonic kidney cell lines (HEK293A) (Thermo Fisher Scientific) were cultured in DMEM supplemented with 10% (vol/vol) fetal bovine serum, penicillin-streptomycin (Life Technologies), GlutaMAX (ThermoFisher Scientific), and Non-Essential Amino Acids (ThermoFisher Scientific). All cell lines were maintained at 37°C in 5% CO_2_. For transient transfection experiments, cells were plated in 10cm plates and transfected the next day using TransIT-293 Transfection Reagent (Mirus) at ~80% confluence. Cells were harvested 36–48 hours after transfection for co-immunoprecipitation and/or western blot analysis. Plasmids used are summarized in S1 Table. Fbw7 knock-down was performed by transducing 293A cells with lentiviruses containing shControl or shFbw7 (CAGAGAAATTGCTTGCTTT), followed by puromycin selection.

### Plasmids and mutagenesis

pCMV24-3xFLAG-Fbw7α wild-type and mutants, pCS2-5xMyc-Fbw7α, pCMV24-3xFLAG-Fbw1 wild-type and mutants, pCS2-HA-SV40 LT, and pCS2-HA-c-Myc have been described [21, 23, 24, 27, 29, 31, 32, 42]. MCPyV T antigens were subcloned into pCS2, and/or pCS2-HA vectors using the In-fusion HD Cloning Kit (Clontech). The pCS2-MCPyV.57kT plasmid was generated by GENEWIZ TurboGENE Gene Synthesis. The MCPyV LT alanine scan mutagenesis was performed by ThermoFisher Scientific GeneArt. MCPyV T antigen mutants were generated using New England BioLabs Q5 Site-Directed Mutagenesis Kit, with primers designed using the NEBase Changer tool (New England Biolabs). pCGN.HA-Fbw7 and pCGN.HA-Fbw7 R465C were provided by Patrick Moore and Yuan Chang (University of Pittsburgh).

### Co-Immunoprecipitation, immunoblotting, and antibodies

Co-immunoprecipitations were performed using a modified protocol from Jianxin You (University of Pennsylvania). Briefly, 293A cells were harvested 36–48 hours after transfection and lysed with NP40 lysis buffer containing cOmplete, EDTA-free Protease Inhibitor Cocktail (Sigma-Aldrich) on ice. Cell lysates were lightly sonicated and the lysates were rotated at 4°C for 1 hour. The BCA Protein Assay Kit (Pierce) was used to quantify the protein concentrations. 500μg of normalized cell lysates were pre-cleared with 25μl of equilibrized Protein A/G Magnetic Beads (Pierce) for 1 hour at 4°C with rotation. The A/G Magnetic Beads were removed from the cellular lysates and discarded, followed by incubation with 10μg of the immunoprecipitation antibody overnight at 4°C with rotation (XT10-donated from Chris Buck, CM2B4 (Santa-Cruz, sc-136172), Ab3-donated from Jim DeCaprio, Ab5-donated from Jim DeCaprio, anti-HA (BioLegend-MMS-101P), 9E10 (made in house), anti-FLAG M2 (Sigma-Aldrich, F1804), or anti-Fbw7 (Bethyl Labs, A301-720). 25μl of protein A/G magnetic beads, per sample, were also blocked in 1% BSA overnight at 4°C with rotation. The next morning, antibody/cellular lysates were added to the 25μl of pre-blocked A/G magnetic beads, and incubated at room temperature, with rotation, for 1 hour. Immunoprecipitated samples were washed several times with KCL buffer, resuspended in SDS sample buffer, boiled, separated by electrophoresis, and transferred to an Immobilon-P PVDF Membrane (Millipore) in parallel with 30μg of whole cell lysate. Membranes were blocked in 4% milk overnight at 4°C, followed by incubation with the primary antibodies described above, in addition to the 2T2 antibody which binds an epitope common to both MCPyV LT and ST (provided by Chris Buck; National Cancer Institute). After washing, a mouse IgG light chain specific HRP conjugated secondary antibody (Cell Signaling-D3V2A, #58802) was incubated with the membranes in 4% milk for one hour followed by washing and chemiluminescent detection with a ChemiDoc Imaging System (Bio-Rad). Actin immunoblotting was performed with **β**-Actin (Cell Signaling-13E5, #5125). c-Myc immunoblotting was also performed (Cell Signaling Technology, D84C12). A more detailed co-immunoprecipitation protocol may be viewed at: dx.doi.org/10.17504/protocols.io.v6ke9cw

### RNA extraction and qRT-PCR analysis

Total RNA was isolated from 293A cells 36–48 hours after transfection using a PureLink RNA Mini Kit (Thermo Fisher Scientific) with DNase I treatment. The SuperScript VILO cDNA Synthesis Kit (Thermo Fisher Scientific) was used to synthesize single-stranded complementary DNA (cDNA) from 1μg total RNA. Fbw7α, and GAPDH expression were evaluated in triplicate using diluted cDNA as template, gene-specific forward and reverse primers (0.3μM), and Power SYBR Green Master Mix (Thermo Fisher Scientific) in an Applied Biosystems StepOnePlus Real-Time PCR system (Thermo Fisher Scientific).

## Supporting information

S1 Table Constructs used throughout the report. Several different constructs were utilized throughout the report and are described above.
(TIF)

S1 Fig The SCF^Fbw7^ ubiquitin ligase complex and its substrates. **(A)** The SCF^Fbw7^ ubiquitin ligase complex binds a target protein through the interaction of the WD40 domain of Fbw7 (green), and the Cdc4 phospho-degron (CPD) of the target substrate. The remaining SCF ubiquitination machinery, including the E2 ubiquitin conjugating enzyme, interacts with Fbw7 via the Fbox domain (yellow) leading to ubiquitination of the substrate protein. The dimerization domain (DD) (pink) mediates the formation of Fbw7 dimers. **(B)** Several proteins contain the conserved Fbw7 phospho-degron sequence (SV40 LT, c-Myc), whereas other proposed domains do not resemble either the Fbw7 or Fbw1 phospho-degron sequences (MCPyV ST LSD, MCPyV LT S239, MCPyV LT S147) [17, 18]. Red and orange residues specify phosphorylated or negatively charged positions within the CPD (SV40 LT has a negatively charged glutamic acid at position +4), and underlined residues depict proposed residues essential for binding [18]. Additional residues important for binding are also colored, such as hydrophobic residues preceding the central phosphorylated threonine (Fbw7-blue), two prolines after the central threonine (Fbw7-green), and the aspartic acid (Fbw1-pink) and glycine (Fbw1-purple) surrounding the central phosphorylated serine. **(C)** It has been proposed that in addition to its normal cellular targets, such as c-Myc, Fbw7 also targets MCPyV LT-t for proteasomal degradation **(C-top panel)**; however, it is proposed that ST, through its Large-T Stabilization Domain (LSD) LSD, is able to bind and sequester Fbw7, thereby reducing turnover of MCPyV LT-t and its other cellular targets **(C-bottom panel)** [17]. **(D)** Due to alternative splicing, the MCPyV T antigens LT, LT-t, 57kT, and ST all contain a shared N-terminal domain (common-T, blue) that is recognized by several antibodies including Ab5 (IP, WB), 2T2 (WB), and XT10 (IP, WB). The MCPyV LT unique region (yellow), shared by LT, LT-t, and 57kT, is recognized by LT specific antibodies CM2B4 (IP, WB) and Ab3 (IP, WB). The MCPyV ST unique region is colored green. IP—immunoprecipitation, WB–western blot.
(TIF)

S2 Fig MCPyV LT-t does not decrease Fbw7 mRNA levels, nor bind Fbw7. **(A)** Fbw7 expression levels when co-expressed with MCPyV LT-t was assessed by qRT-PCR. **(B)** 293A cells were transfected with individual or combinations of Fbw7 (4.5μg), HA-SV40 LT (5μg), HA-SV40 LT-T701A (5μg), or MCPyV LT-t (10.5μg). For the final 12 hours before harvesting, the cells were treated with 10μM MG132. Both MCPyV and SV40 LT proteins were pulled-down with XT10, and immunoblotted with anti-FLAG.
(TIF)

S3 Fig Additional MCPyV T antigen specific immunoprecipitation antibodies reveal a unidirectional interaction between MCPyV T antigens and Fbw7. **(A, B)** A co-immunoprecipitation between MCPyV T antigens (LT, LT-t, LT S239A, ST, ST ΔLSD) and Fbw7 (wild-type and R505L mutant) was performed through pull-down of an antibody recognizing **(A)** common-T (Ab5) or **(B)** LT (CM2B4 or Ab3). Co-immunoprecipitated Fbw7 was detected by immunoblotting with anti-FLAG. MCPyV T antigens were detected with 2T2 immunoblotting. Asterisks (*) denote non-specific bands.
(TIF)

S4 Fig Identification of the domain of MCPyV LT/57kT responsible for binding Fbw7α. **(A)** MCPyV LT, 57kT, and ST, but not LT-t, co-immunoprecipitate Fbw7α after pull-down of the T antigens. This suggests the domain responsible for interacting with Fbw7 on the T antigens is not shared with LT-t (red), but found on the C-terminal 100 amino acids of LT and 57kT (green), or ST unique region (blue). **(B-E)** An alanine scan of MCPyV LT/57kT was performed on the C-terminal 100 amino acids in which sequential 5 amino acid alanine substitutions were created and tested for their ability to co-immunoprecipitate Fbw7. 293A cells were transfected with individual or combinations of Fbw7 (4.5μg), MCPyV LT-t (10.5μg), or MCPyV wild-type LT or alanine scan mutants (1–20) (5μg), followed by pull-down of MCPyV LT by XT10, and immunoblotting with an anti-FLAG antibody.
(TIF)

S5 Fig MCPyV T antigens bind to an unidentifiable domain within the shared region of Fbw7 isoforms. **(A)** To assess whether MCPyV T antigens recognize the Fbw7α isoform specific N-terminus (blue), or the C-terminal common region shared by all Fbw7 isoforms (orange), several constructs were tested in their ability to co-immunoprecipitate with MCPyV T antigens. Fbw7 ΔN encodes only the C-terminal common region found in all Fbw7 isoforms. α70x encodes the Fbw7α isoform specific N-terminus, in addition to 70 amino acids of the common region. Fbw7α ΔC encodes only the Fbw7α isoform specific N-terminus. Whether the dimerization, Fbox, and WD40 domains are retained in each construct is depicted. **(B)** 293A cells were transfected with 4.5μg of either wild-type or mutant Fbw7 (described in S5A), all of which are FLAG tagged, and/or MCPyV LT (5μg), or MCPyV LT-t (10.5μg). MCPyV LT and LT-t were pulled-down from the whole cell lysate using XT10, and immunoblotted with anti-FLAG. Fbw7α ΔC did not express. **(C)** An ΔFbox and dimerization domain double mutant (Fbw7 ΔFD) (3μg) was also assessed in its ability to co-immunoprecipitate with MCPyV LT and ST.
(TIF)

S6 Fig Additional MCPyV T antigen specific immunoprecipitation antibodies reveal a unidirectional interaction between MCPyV LT, ST, and Fbw1. **(A, B)** A co-immunoprecipitation between MCPyV T antigens (LT, LT-t, LT S147A, ST) and Fbw1 was performed through pull-down of an antibody recognizing **(A)** common-T (Ab5) or **(B)** LT (CM2B4 and Ab3). Co-immunoprecipitated Fbw1 was detected by immunoblotting with anti-FLAG. Asterisks (*) denote non-specific bands.
(TIF)
