# Supplementary figures and images for: Merkel cell polyomavirus Tumor antigens expressed in Merkel cell carcinoma function independently of the ubiquitin ligases Fbw7 and β-TrCP
Source: PLoS Pathog. 2019 Jan 28;15(1):e1007543. doi: 10.1371/journal.ppat.1007543 (PMC6366716; doi:10.1371/journal.ppat.1007543)

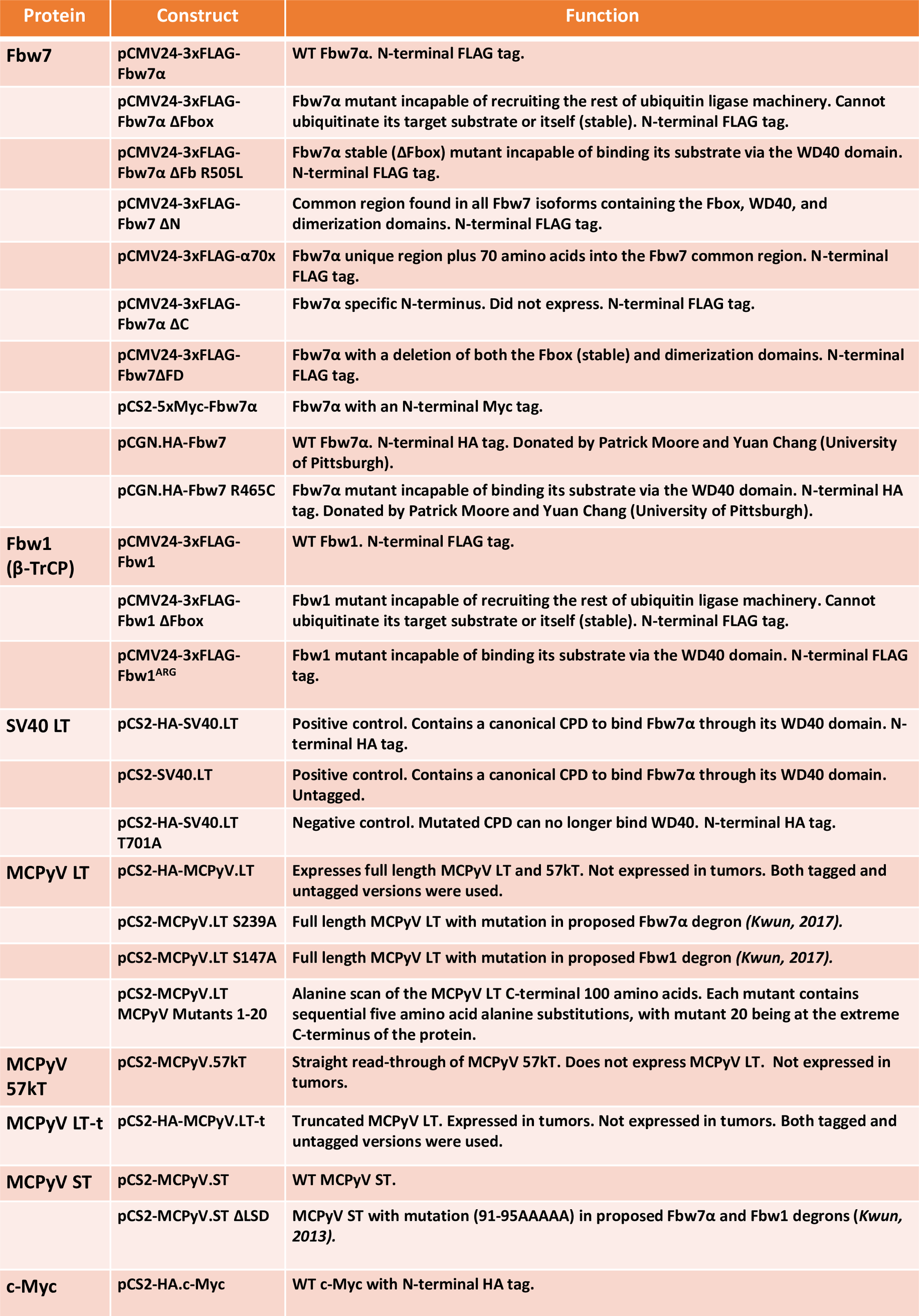

Supplement: S1 Table — Several different constructs were utilized throughout the report and are described above. (TIF) [file ppat.1007543.s001.tif]

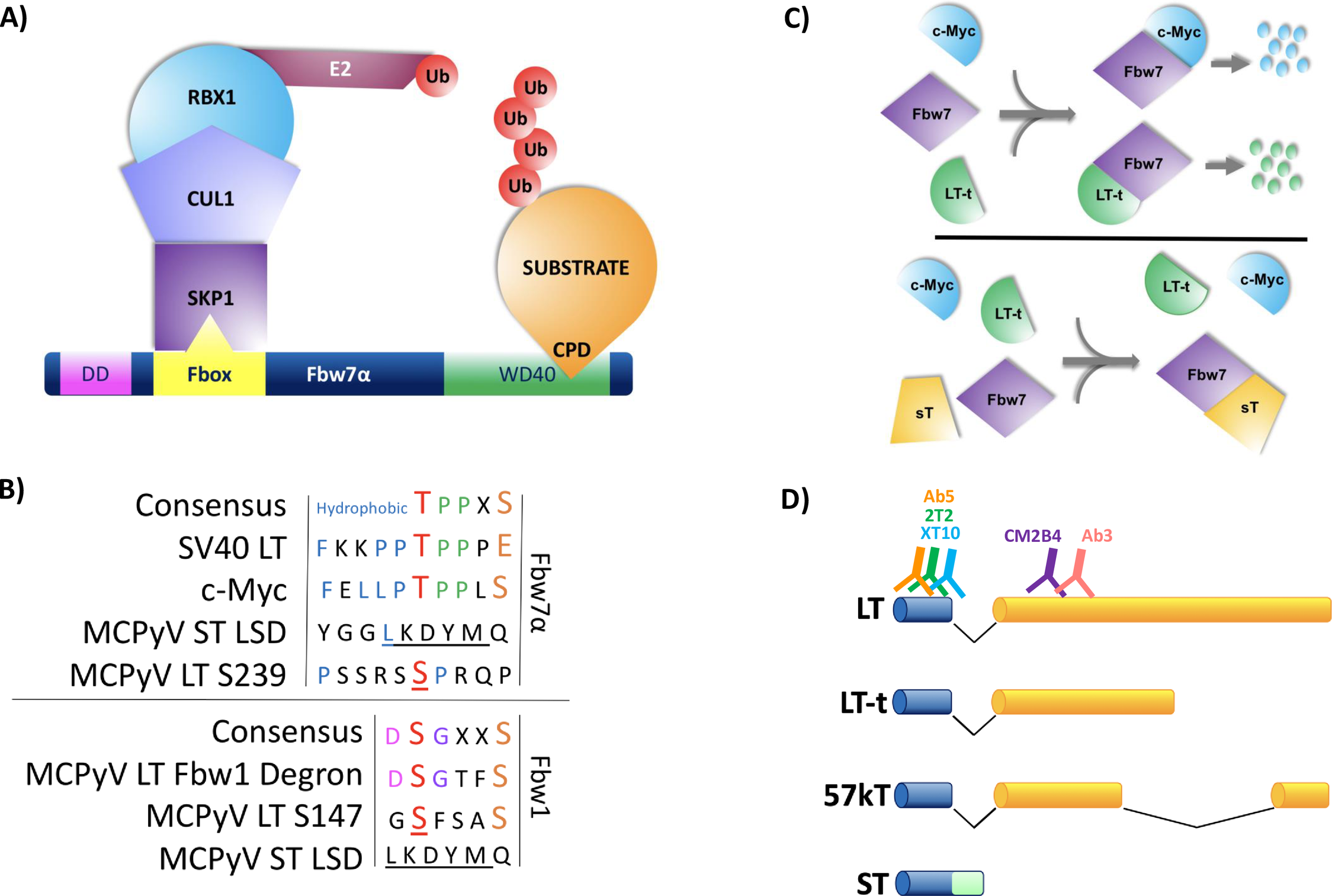

Supplement: S1 Fig — (A) The SCFFbw7 ubiquitin ligase complex binds a target protein through the interaction of the WD40 domain of Fbw7 (green), and the Cdc4 phospho-degron (CPD) of the target substrate. The remaining SCF ubiquitination machinery, including the E2 ubiquitin conjugating enzyme, interacts with Fbw7 via the Fbox domain (yellow) leading to ubiquitination of the substrate protein. The dimerization domain (DD) (pink) mediates the formation of Fbw7 dimers. (B) Several proteins contain the conserved Fbw7 phospho-degron sequence (SV40 LT, c-Myc), whereas other proposed domains do not resemble either the Fbw7 or Fbw1 phospho-degron sequences (MCPyV ST LSD, MCPyV LT S239, MCPyV LT S147) [17, 18]. Red and orange residues specify phosphorylated or negatively charged positions within the CPD (SV40 LT has a negatively charged glutamic acid at position +4), and underlined residues depict proposed residues essential for binding [18]. Additional residues important for binding are also colored, such as hydrophobic residues preceding the central phosphorylated threonine (Fbw7-blue), two prolines after the central threonine (Fbw7-green), and the aspartic acid (Fbw1-pink) and glycine (Fbw1-purple) surrounding the central phosphorylated serine. (C) It has been proposed that in addition to its normal cellular targets, such as c-Myc, Fbw7 also targets MCPyV LT-t for proteasomal degradation (C-top panel); however, it is proposed that ST, through its Large-T Stabilization Domain (LSD) LSD, is able to bind and sequester Fbw7, thereby reducing turnover of MCPyV LT-t and its other cellular targets (C-bottom panel) [17]. (D) Due to alternative splicing, the MCPyV T antigens LT, LT-t, 57kT, and ST all contain a shared N-terminal domain (common-T, blue) that is recognized by several antibodies including Ab5 (IP, WB), 2T2 (WB), and XT10 (IP, WB). The MCPyV LT unique region (yellow), shared by LT, LT-t, and 57kT, is recognized by LT specific antibodies CM2B4 (IP, WB) and Ab3 (IP, WB). The MCPyV ST unique [file ppat.1007543.s002.tif]

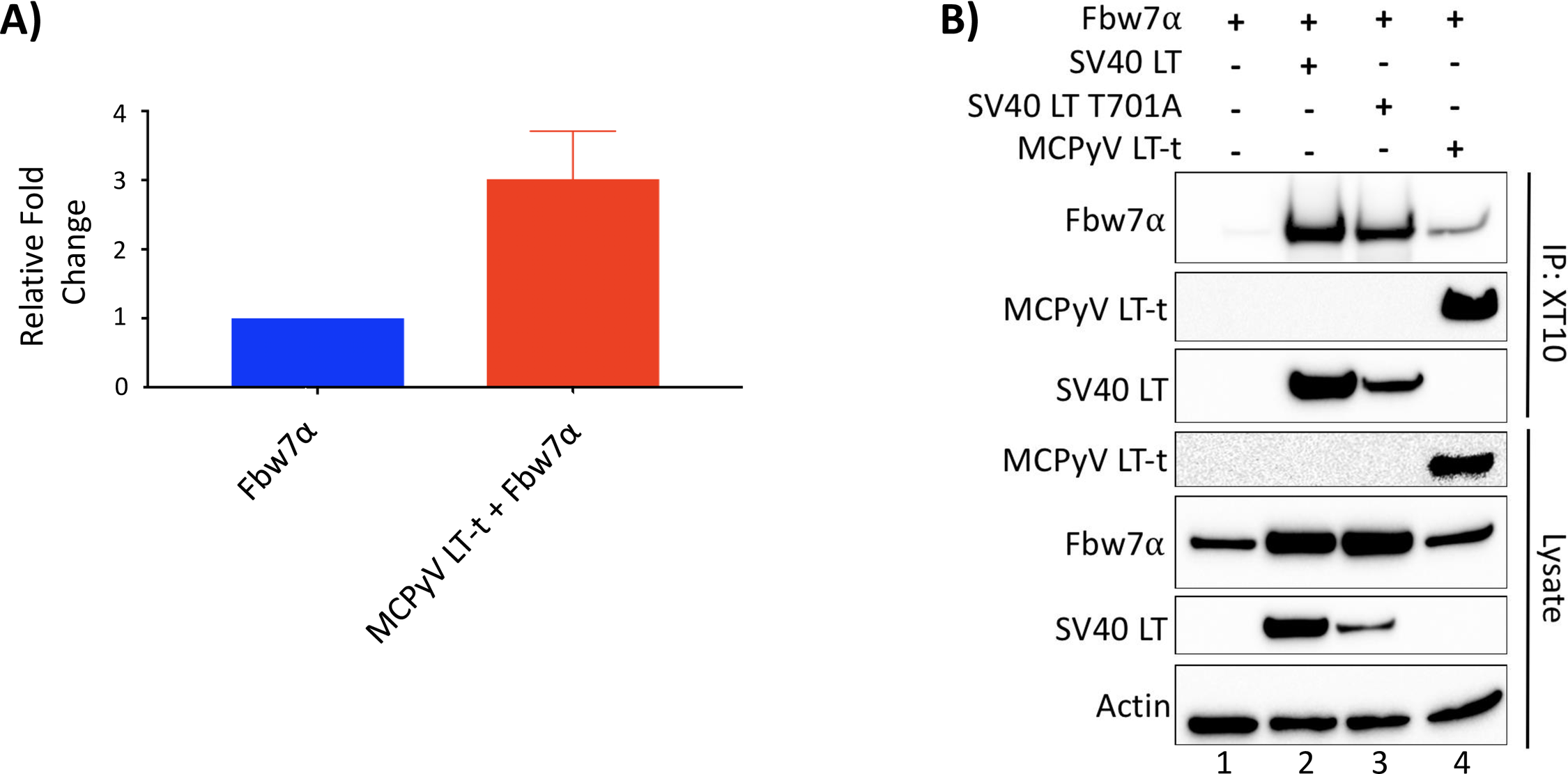

Supplement: S2 Fig — (A) Fbw7 expression levels when co-expressed with MCPyV LT-t was assessed by qRT-PCR. (B) 293A cells were transfected with individual or combinations of Fbw7 (4.5μg), HA-SV40 LT (5μg), HA-SV40 LT-T701A (5μg), or MCPyV LT-t (10.5μg). For the final 12 hours before harvesting, the cells were treated with 10μM MG132. Both MCPyV and SV40 LT proteins were pulled-down with XT10, and immunoblotted with anti-FLAG. (TIF) [file ppat.1007543.s003.tif]

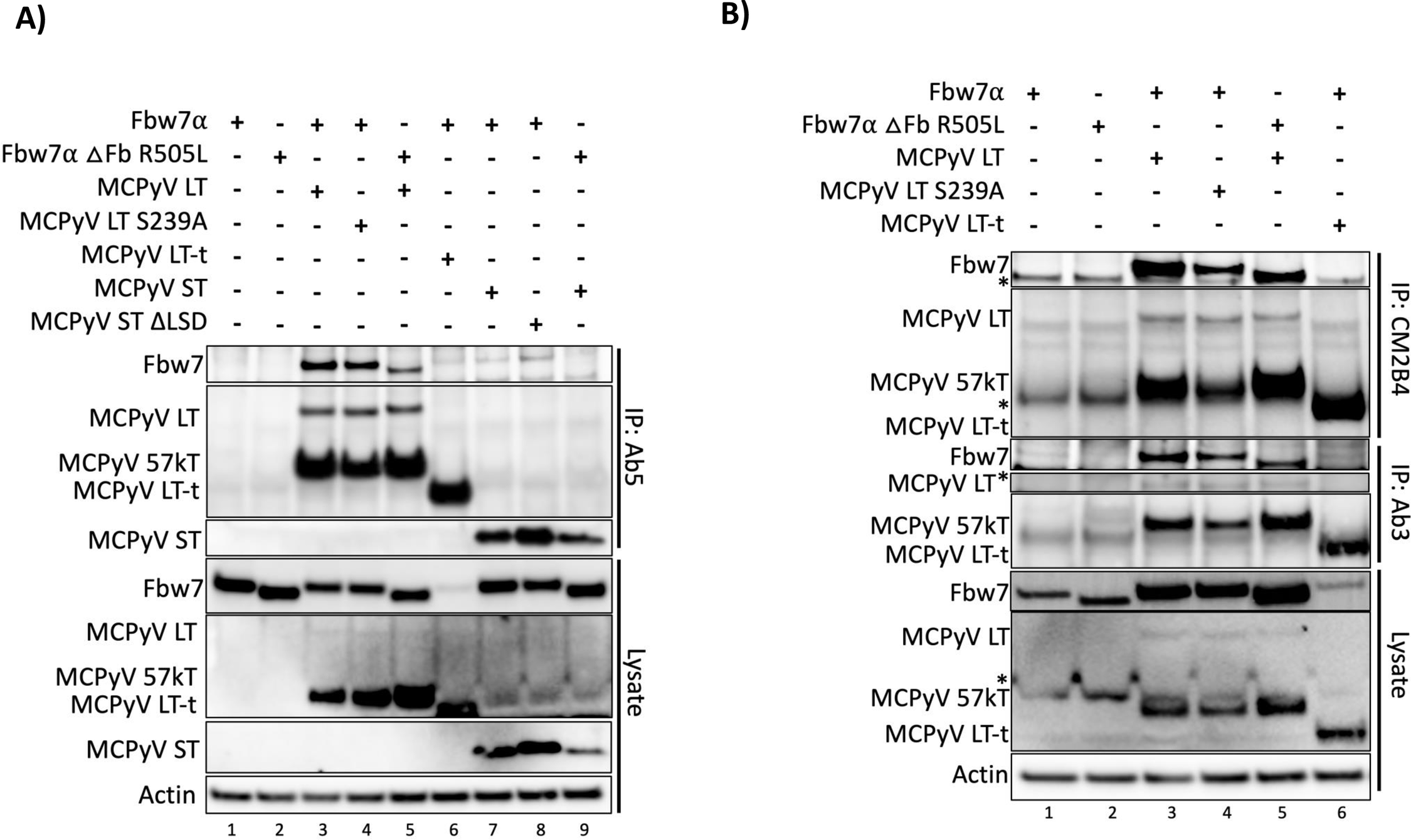

Supplement: S3 Fig — (A, B) A co-immunoprecipitation between MCPyV T antigens (LT, LT-t, LT S239A, ST, ST ΔLSD) and Fbw7 (wild-type and R505L mutant) was performed through pull-down of an antibody recognizing (A) common-T (Ab5) or (B) LT (CM2B4 or Ab3). Co-immunoprecipitated Fbw7 was detected by immunoblotting with anti-FLAG. MCPyV T antigens were detected with 2T2 immunoblotting. Asterisks (*) denote non-specific bands. (TIF) [file ppat.1007543.s004.tif]

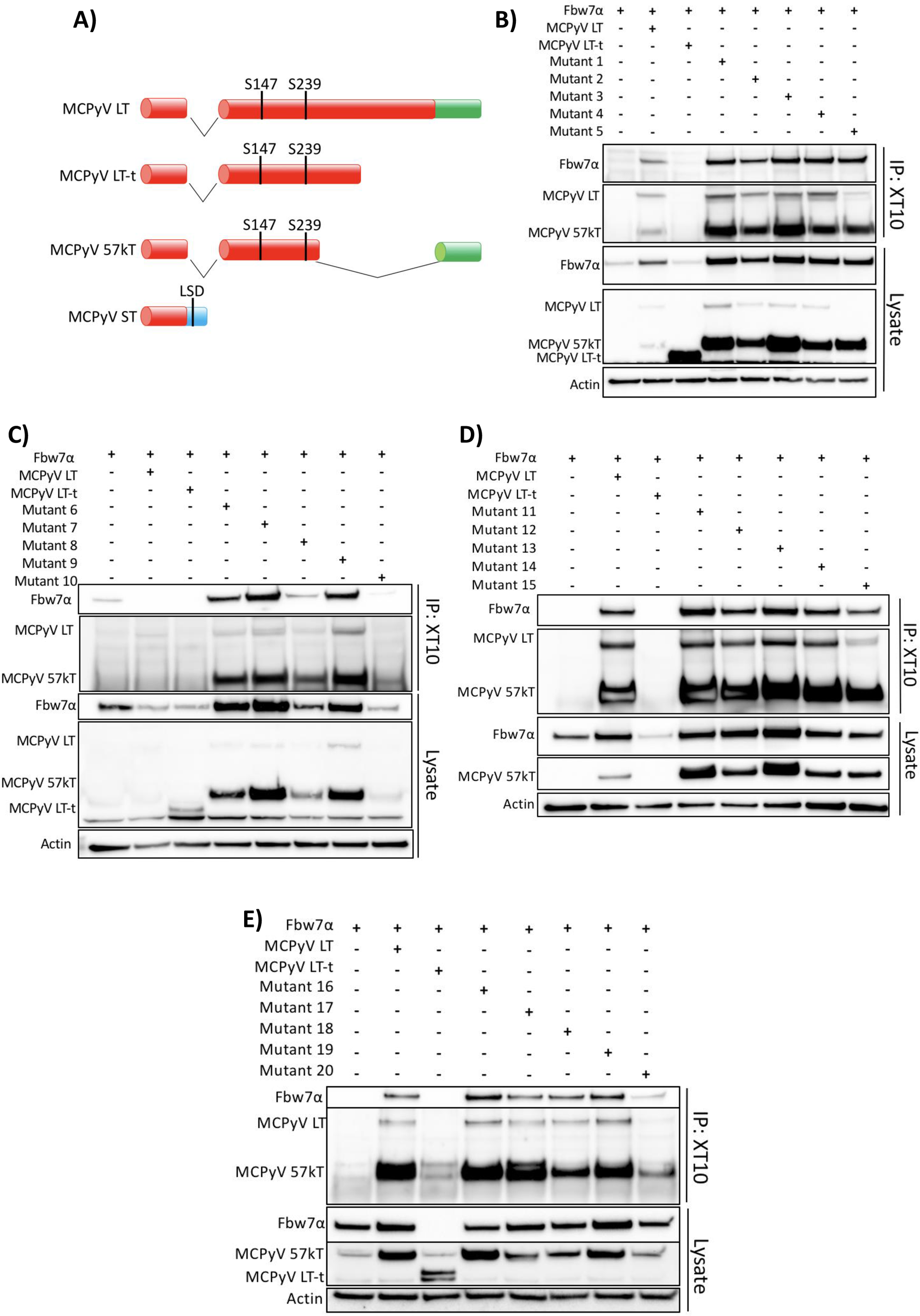

Supplement: S4 Fig — (A) MCPyV LT, 57kT, and ST, but not LT-t, co-immunoprecipitate Fbw7α after pull-down of the T antigens. This suggests the domain responsible for interacting with Fbw7 on the T antigens is not shared with LT-t (red), but found on the C-terminal 100 amino acids of LT and 57kT (green), or ST unique region (blue). (B-E) An alanine scan of MCPyV LT/57kT was performed on the C-terminal 100 amino acids in which sequential 5 amino acid alanine substitutions were created and tested for their ability to co-immunoprecipitate Fbw7. 293A cells were transfected with individual or combinations of Fbw7 (4.5μg), MCPyV LT-t (10.5μg), or MCPyV wild-type LT or alanine scan mutants (1–20) (5μg), followed by pull-down of MCPyV LT by XT10, and immunoblotting with an anti-FLAG antibody. (TIF) [file ppat.1007543.s005.tif]

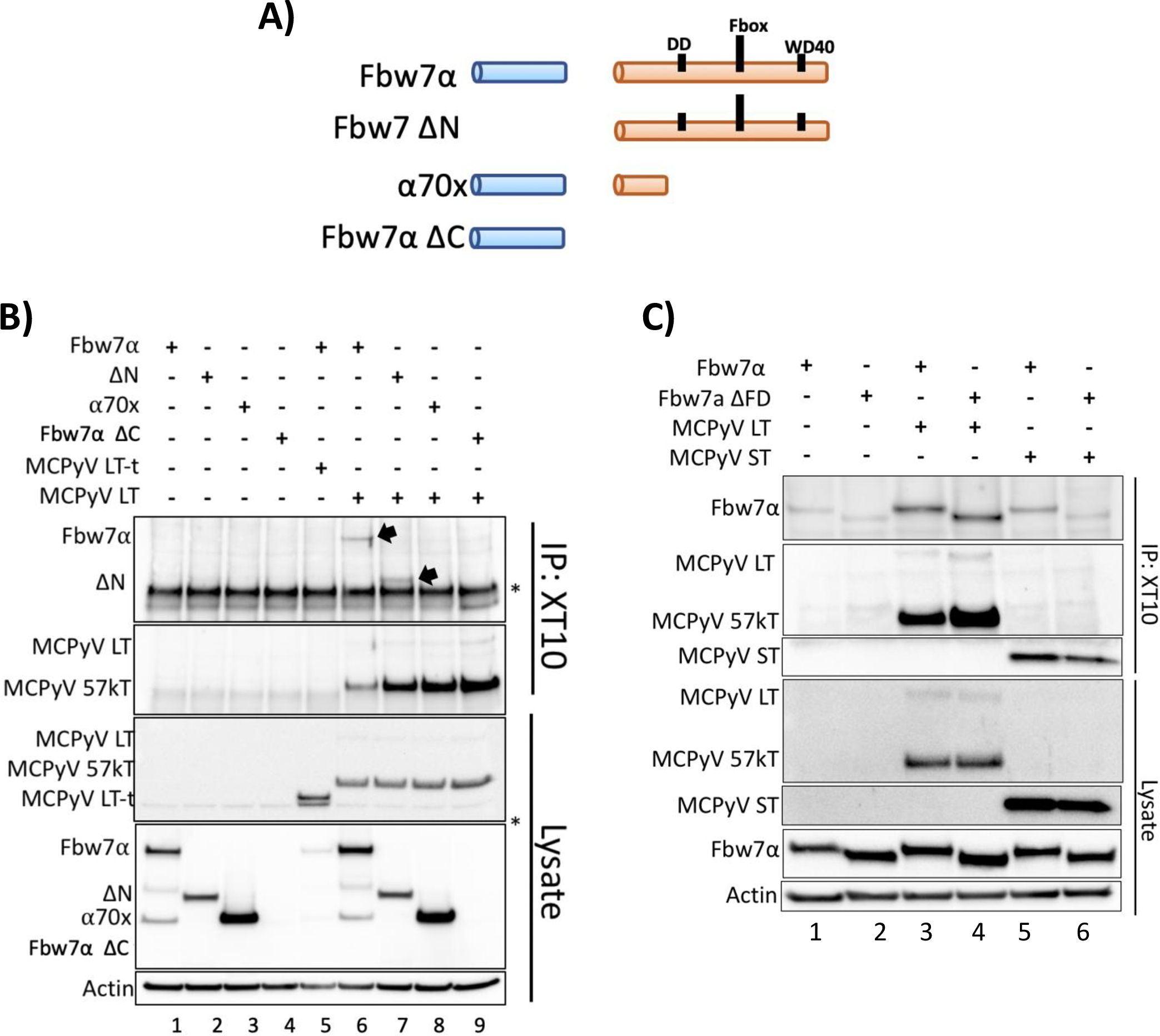

Supplement: S5 Fig — (A) To assess whether MCPyV T antigens recognize the Fbw7α isoform specific N-terminus (blue), or the C-terminal common region shared by all Fbw7 isoforms (orange), several constructs were tested in their ability to co-immunoprecipitate with MCPyV T antigens. Fbw7 ΔN encodes only the C-terminal common region found in all Fbw7 isoforms. α70x encodes the Fbw7α isoform specific N-terminus, in addition to 70 amino acids of the common region. Fbw7α ΔC encodes only the Fbw7α isoform specific N-terminus. Whether the dimerization, Fbox, and WD40 domains are retained in each construct is depicted. (B) 293A cells were transfected with 4.5μg of either wild-type or mutant Fbw7 (described in S5A), all of which are FLAG tagged, and/or MCPyV LT (5μg), or MCPyV LT-t (10.5μg). MCPyV LT and LT-t were pulled-down from the whole cell lysate using XT10, and immunoblotted with anti-FLAG. Fbw7α ΔC did not express. (C) An ΔFbox and dimerization domain double mutant (Fbw7 ΔFD) (3μg) was also assessed in its ability to co-immunoprecipitate with MCPyV LT and ST. (TIF) [file ppat.1007543.s006.tif]

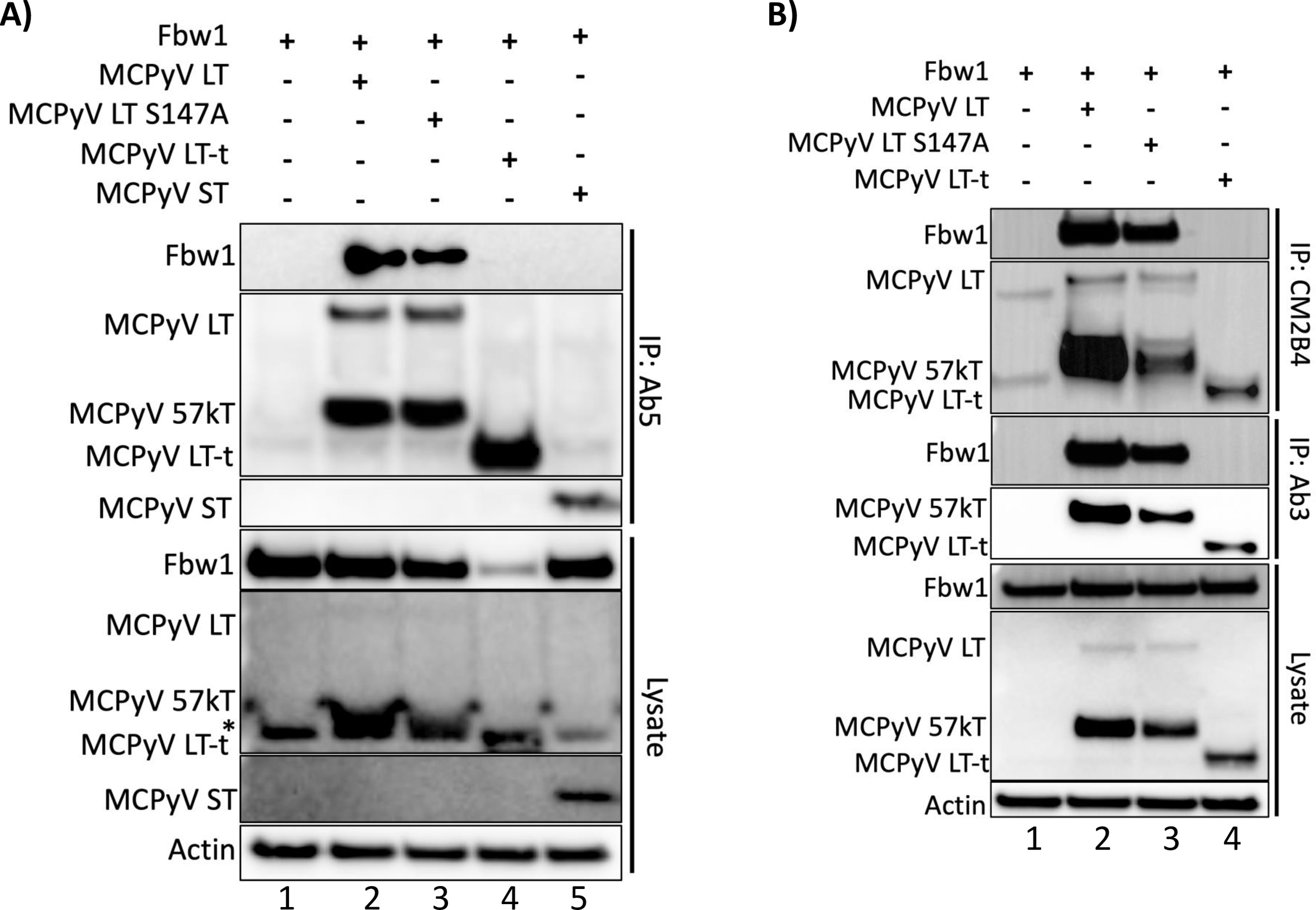

Supplement: S6 Fig — (A, B) A co-immunoprecipitation between MCPyV T antigens (LT, LT-t, LT S147A, ST) and Fbw1 was performed through pull-down of an antibody recognizing (A) common-T (Ab5) or (B) LT (CM2B4 and Ab3). Co-immunoprecipitated Fbw1 was detected by immunoblotting with anti-FLAG. Asterisks (*) denote non-specific bands. (TIF) [file ppat.1007543.s007.tif]
